# GDF15 in Patients with Autoimmune Primary Adrenal Insufficiency

**DOI:** 10.3390/ijms27052260

**Published:** 2026-02-27

**Authors:** Aleksandra Zdrojowy-Wełna, Jowita Halupczok-Żyła, Katarzyna Kolačkov, Justyna Kuliczkowska-Płaksej, Aleksandra Jawiarczyk-Przybyłowska, Barbara Stachowska, Iga Zendran-Zahorska, Marek Bolanowski

**Affiliations:** 1 Department of Endocrinology and Internal Medicine, Wroclaw Medical University, 50-367 Wroclaw, Polandmarek.bolanowski@umw.edu.pl (M.B.); 2 Department of Pathology, Wroclaw Medical University, 50-367 Wroclaw, Poland; 3 Laboratory of Molecular Endocrinology, Department of Endocrinology and Internal Diseases, Wroclaw Medical University, 50-367 Wroclaw, Poland

**Keywords:** primary adrenal insufficiency, Addison’s disease, GDF15, autoimmune adrenal insufficiency

## Abstract

Growth differentiation factor 15 (GDF15) is a stress-response protein that conveys cellular distress signals to the brain and activates neural pathways leading to weight loss. GDF15 levels are increased in glucocorticoid deficiency; however, multiple factors may influence its levels in patients with primary adrenal insufficiency (PAI). The objective of this study was to determine circulating GDF15 levels in patients with PAI compared with a control group and to assess their associations with other clinical parameters. We included 37 patients (22 females) with autoimmune PAI and 47 healthy controls. Serum GDF15 levels, together with anthropometrical, hormonal and biochemical parameters, were assessed. Patients with PAI had significantly higher circulating GDF15 levels than controls did (1276.8 ± 952.1 vs. 682.8 ± 270.2 pg/mL, *p* < 0.001). In both groups, GDF15 levels were positively correlated with age (*p* < 0.001). In patients with PAI, GDF15 showed positive correlations with disease duration and duration of autoimmune thyroid disease, gonadotropin levels, waist-to-hip ratio, and body fat percentage, and negative correlations with DHEAS and sex hormone levels. In conclusion, GDF15 levels are increased in patients with PAI compared with healthy controls and correlate with age and the duration of autoimmune disease.

## 1. Introduction

Growth differentiation factor 15 (GDF15) is a member of the transforming growth factor beta (TGFβ) superfamily [1]. It is considered a stress-response cytokine because its levels increase in many physiological and pathological states, such as pregnancy, aging, exercise, chronic inflammatory disease, renal failure, cardiac failure, neoplasia, and cytotoxic chemotherapy [2]. GDF15 may be secreted by a wide range of cell types in response to a variety of stressors; however, particular interest relates to its central action on energy homeostasis.

The GDF15 receptor is a glial cell-derived neurotrophic factor family receptor alpha-like (GFRAL)–RET heterodimer, of which GFRAL is expressed exclusively in the hindbrain, and its activation leads to reduced food intake and loss of body weight [3]. This effect is independent of other hormones regulating food intake [4]. In mice, overexpression of GDF15 results in lower energy intake, whereas GDF15 knockout mice exhibit increased energy intake and body mass; these effects are mediated centrally in the hindbrain [5,6,7]. Moreover, GDF15 administration in mice triggers conditioned taste aversion [8]. It is likely that the role of GDF15 is to act as an endocrine signal conveying somatic distress to the brain [3]. It has been shown that metformin increases circulating levels of GDF15 in humans, which is crucial for its effect on lowering body weight [9]. In many diseases, including cardiac failure, chronic kidney disease, and neoplasms, GDF15 has been linked to the development of cachexia [5,10,11,12]. The exact mechanism by which GDF15 predisposes to weight loss remains unclear. Some authors suggest that, evolutionarily, GDF15 served as an alarm signal indicating toxin ingestion, which may have been particularly important in pregnant females to protect the fetus from teratogens [3]. This hypothesis is supported by evidence that GDF15 plays a causal role in nausea and vomiting during pregnancy [13].

Primary adrenal insufficiency (PAI) is a rare endocrine condition, with an estimated prevalence of 100–140 cases per million [14]. The disease is characterized by inadequate production of glucocorticoids and mineralocorticoids by the adrenal glands, and despite hormonal substitution, patients with PAI experience a 2.5-fold increase in mortality compared with the healthy population [15]. Some symptoms of PAI, such as weight loss, nausea, and vomiting, resemble the effects of GDF15. Therefore, it has been hypothesized that GDF15 levels are increased in patients with PAI. This was confirmed in a study by Melvin et al., in which 18 patients with Addison’s disease and 9 patients with congenital adrenal hyperplasia had higher circulating GDF15 levels than controls, and intravenous hydrocortisone replacement reduced GDF15 levels in patients with PAI [16]. However, to the best of our knowledge, there are no studies assessing correlations between GDF15 and other metabolic parameters and disease-related factors in patients with PAI.

Therefore, the aim of our study was to evaluate circulating GDF15 levels in a homogeneous group of patients with autoimmune PAI in comparison with healthy controls and to correlate GDF15 levels with anthropometric, metabolic, and hormonal parameters in both groups.

## 2. Results

### 2.1. General Characteristics of Patients and Controls

The general characteristics of the 37 patients with PAI (22 females, 59.5%) and the control group are presented in Table 1; the mean age of the PAI group was 48.9 ± 13.1 years, and the mean body mass index (BMI) was 25.4 ± 4.2 kg/m^2^, with no significant differences compared with controls (*p* = 0.413 for sex, *p* = 0.207 for age, *p* = 0.113 for BMI). Additional characteristics of patients with PAI are shown in Table 2. The mean disease duration was 11.3 ± 12.7 years, with mean daily doses of 26.3 ± 5.8 mg hydrocortisone and 0.06 ± 0.06 mg fludrocortisone. Electrolyte levels were within the normal range.

### 2.2. Differences Between GDF15, Hormonal, and Metabolic Parameters Between Patients and Controls

Patients with PAI had significantly higher GDF15 levels than the controls did (1276.8 ± 952.1 vs. 682.8 ± 270.2 pg/mL, *p* < 0.001). They also exhibited higher levels of adrenocorticotropin (ACTH) and triglycerides, and lower levels of dehydroepiandrosterone sulfate (DHEAS) compared with controls (*p* < 0.001 for all). Other metabolic parameters, including cholesterol levels, fasting glucose, the Homeostatic Model Assessment of Insulin Resistance (HOMA-IR), and the percentage of fat mass measured by total-body dual-energy X-ray absorptiometry (DXA), did not differ between patients and controls.

### 2.3. Correlations Between GDF15 and Significant Clinical Factors in Patients with Adrenal Insufficiency and Controls

In both patients with PAI and controls, GDF15 levels correlated significantly with age (Table 3 and Table 4).

In the PAI group, GDF15 levels were positively correlated with the duration of PAI, the presence of autoimmune thyroid disease, gonadotropin levels, waist-to-hip ratio, and the percentage of body fat measured by DXA. Negative correlations were observed with DHEAS and sex hormone levels (Table 3). We did not observe correlations between GDF15 and doses of hydrocortisone and fludrocortisone, urinary cortisol excretion, or ACTH levels. In controls, among all metabolic parameters only fasting glucose was significantly positively correlated with GDF15, whereas insulin-like growth factor 1 (IGF-1) was negatively correlated with GDF15 (Table 4).

The results of GDF15 levels in patients with PAI are presented in Supplementary Material (Table S1).

## 3. Discussion

Our data show that in the group of patients with autoimmune PAI, GDF15 levels were significantly higher than in the age-, sex-, and BMI-matched control group and correlated with the duration of autoimmune disease. In patients with PAI, GDF15 levels were positively correlated with the percentage of fat mass, waist-to-hip ratio (WHR), and gonadotropin levels, and negatively correlated with sex hormones and DHEAS levels. Older age was associated with higher GDF15 levels in both patients with PAI and controls.

Consistent with our results, one study has shown that patients with glucocorticoid deficiency exhibit elevated levels of circulating GDF15. However, Melvin et al. performed short-term deprivation of glucocorticoid substitution prior to measuring GDF15, while subsequent hydrocortisone replacement led to a decrease in GDF15 levels, which was dose-dependent [16].

Authors concluded that glucocorticoids modulate GDF15 levels by reducing the expression of the GDF15 gene [17]. Secondly, glucocorticoids have significant antiemetic potential, while factors related to increased risk of nausea and vomiting, like radiotherapy and chemotherapy, significantly increase GDF15 levels [3,18]. In contrast to the study by Melvin et al., patients in our study continued their glucocorticoid and mineralocorticoid supplementation during GDF15 assessment. This shows that despite long-lasting treatment, which should mimic the physiological adrenal function, GDF15 levels remained increased. We are aware of the fact that clinical evaluation of hydrocortisone supplementary doses may be difficult and that standard replacement therapy in PAI does not correspond with physiological cortisol dynamics. Therefore, subclinical glucocorticoid deficiency in PAI patients may have led to increased GDF15 levels in our study. However, the mean daily hydrocortisone dose used by these patients was 26.3 ± 5.8 mg, which is considered rather high; we also did not notice any clinical signs or electrolytes deviations suggestive of adrenal insufficiency. We also did not observe any correlations between GDF15 levels and patient-reported daily doses of hydrocortisone and fludrocortisone, or with urinary cortisol excretion. Only the negative correlation between DHEAS and GDF15 in our study supports the hypothesis that higher GDF15 is primarily related to glucocorticoid deficiency, while the remnant adrenal steroidogenesis reduces the circulating GDF15 in patients with PAI.

Another possible explanation for increased GDF15 in PAI patients on stable glucocorticoid replacement therapy is the fact that PAI is a chronic disease placing a heavy burden and stress on patients, and GDF15 is a stress response cytokine [8]. This is supported by the positive correlation between GDF15 and the duration of autoimmune PAI and thyroid disease. There is substantial data that despite stable replacement therapy, patients with PAI suffer from impaired quality of life [19,20]. Interestingly, we have previously shown that in patients with PAI, worse health-related quality of life was associated with older age, female sex and higher gonadotropin levels; also, fatigue scores were worse in the presence of autoimmune thyroiditis [21]. In line with those results, in the current study, GDF15 correlated with age, gonadotropins, the duration of PAI and autoimmune thyroiditis. This suggests that GDF15 correlates with prolonged exposure to pathogenic stress factors. Recently, the allostatic load (AL) has been attracting more attention, defined as the occurrence of adverse changes in the body in response to the chronic effects of multiple stressors over a lifetime [22]. It has been shown that endocrine diseases are related to increased allostatic load, although no such studies have been performed in patients with PAI [23]. This area warrants further investigation in patients with PAI, and GDF15 may correspond with allostatic load.

Other authors have shown that GDF15 is also correlated with frailty [24]. Frailty is a state of vulnerability to poor resolution of homeostasis after a stressor, as a consequence of cumulative decline in many physiological systems, which strongly resembles the aforementioned patients with high allostatic loads. Also, frailty is associated with sarcopenia, defined as a skeletal muscle disorder characterized by accelerated loss of muscle function and mass [25]. In our group of patients with PAI, GDF15 correlated with the percentage of body fat and central obesity (WHR), while in healthy controls GDF15 correlated with fasting glucose. This may be secondary to age-related changes in body composition, where typically weight loss is caused by a decrease in muscle mass, and may be accompanied with an increased percentage of fat mass [25]. This phenotype is related to increased cardiovascular risk despite normal body weight [26]. In a case-controlled study, diabetic individuals had a significantly higher GDF15 serum level compared to pre-diabetic and healthy groups. Moreover, GDF15 was measured in visceral adipose tissue, where it was also elevated in diabetic subjects compared to controls [27]. In another study, increased circulating GDF15 was correlated with the risk of lower extremity atherosclerotic disease in patients with type 2 diabetes, and this relationship was independent of BMI [28]. In patients with severe obesity, GDF15 was strongly associated with beta cell function parameters [29]. However, the role of GDF15 in cardiometabolic health is not clear and its therapeutic potential has also been also considered, particularly due to its negative impact on food intake [30]. It has to be underlined, however, that no clear association between GDF15 and unfavorable body composition can be concluded from our study and other studies so far, because age is a strong confounder of all these correlations. In our study group, older age was correlated with increased GDF15 levels both in patients with PAI and in healthy controls, showing that this association is not disease-dependent. This result stays in line with other studies; for example, in a cohort of over 600 individuals aged 21–113, GDF15 was significantly associated with age and its level was inversely correlated with survival in the oldest subjects [31]. In a longitudinal analysis, GDF15 levels increased by 11% on average after 5 years of follow-up [32]. It is believed that age-related inflammatory, metabolic, and oxidative stresses may increase the expression of GDF15 [33]. Indeed, more prospective randomized studies are needed to elucidate the diverse pathophysiological roles of GDF15 in cardiometabolic disorders, as well as its physiological functions across the life course.

GDF15 was positively correlated with gonadotropins and negatively with sex hormones and IGF-1 in patients with PAI. To our best knowledge, this has not been reported before. In our study group, aged between 24 and 73 years, these correlations may be secondary to age-related loss of gonadal function, a physiological decrease in IGF-1 and an increase in gonadotropin levels. As we discussed above, the association between GDF15 and age has been confirmed in our and many other studies; therefore, the correlation with gonadotropins and IGF-1 may be only related to age [31,32,33]. However, the cluster of correlations between GDF15 and increased fat mass, lower sex hormones and lower IGF-1 suggests correlation between GDF15 and an unfavorable body composition with decreased muscle mass and increased cardiovascular risk. Further prospective, randomized studies are needed to assess if GDF15 is the cause or an effect of increased cardiovascular risk, or if it is only a reflection of age-related metabolic changes.

Our study had several limitations. First, the observational nature of this study prevented us from drawing conclusions about the causal relationships between GDF15 and the parameters evaluated in our study. Secondly, the number of patients may not be adequate to discover associations between GDF15 and other clinical factors in patients with PAI, which is a consequence of the rarity of PAI. Also, evaluation of a wider range of inflammatory markers (like IL-6, IFN, and TNFα) may have given additional information on the immunosuppressive role of GDF15 in patients with PAI. We only examined C-reactive protein levels, which did not correlate with GDF15. The immunomodulatory role of GDF15 was also suggested, because GDF15 showed anti-inflammatory action in myocardial infarction, sepsis and neoplasm in mice studies [3]. However, our study was not designed to explore the immunosuppressive effect of GDF15 in patients with PAI; this area remains an interesting field for further studies. Despite these limitations, our study comprises the largest number of patients with PAI who underwent complex evaluation of GDF15 levels together with a wide range of other biochemical, hormonal and anthropometrical parameters. Therefore, we believe that our results bring important information about the possible role of GDF15 in patients with PAI, which should be a premise to further studies in this area.

In conclusion, in a group of patients with autoimmune, long-lasting PAI, GDF15 levels were significantly higher than in healthy controls, and correlated with the duration of autoimmune endocrinopathies. GDF15 was significantly associated with age in patients with PAI and in controls. There were significant correlations between GDF15 and parameters related to unfavorable body composition, like the percentage of body fat, waist-to-hip ratio, and lower sex hormones in patients with PAI, and with fasting glucose and lower IGF-1 in healthy controls. However, this may be only secondary to the association between GDF15 and age, and the design of our study prevents us from drawing conclusions about the causality of this effect. Further studies are needed to clarify the exact role of GDF15 in patients with PAI.

## 4. Materials and Methods

### 4.1. Subjects

We included 37 patients diagnosed with autoimmune PAI (59.5% women; mean age 48.9 ± 13.1 years) and 47 sex-, age-, and BMI-matched control group study participants (68.1% women; mean age 45.9 ± 13.7 years). Patients were treated at the Department of Endocrinology and Internal Diseases in Wrocław, Poland, between February 2020 and February 2024.

The diagnosis of PAI was based on the Endocrine Society Clinical Guidelines criteria [14]. The inclusion criterion was a diagnosis of autoimmune PAI treated with hydrocortisone and fludrocortisone for at least 3 months. The autoimmune cause of adrenal insufficiency was confirmed on the basis of positive testing for autoantibodies against adrenal 21 hydroxylase or the coexistence of other autoimmune diseases, without any other known cause of adrenal insufficiency. The exclusion criteria were a duration of hormonal substitution therapy shorter than 3 months in the group of patients with adrenal insufficiency, ischemic heart disease, chronic kidney disease, acute infection within the last 4 weeks, and neoplastic disease in the patient’s history.

In the group with PAI, 20 patients (54%) had primary autoimmune hypothyroidism treated with L-thyroxine; among this group 5 patients (13.5%) had also type 1 diabetes treated with insulin. Therefore, 20 patients (54%) had autoimmune polyglandular syndrome (APS) type 2, while 17 (46%) patients had isolated PAI.

Nine patients (24%) had hypercholesterolemia treated with statins.

Four females in the adrenal insufficiency group and no female in the control group received estro-progestogens as postmenopausal substitution therapy. In these patients, estradiol levels were not included in the analysis.

All patients had normal kidney and liver function.

### 4.2. Clinical and Laboratory Examinations

- Subjective and physical examination

A thorough medical history was taken, including detailed information about hormonal substitution (current doses and mean doses for the last 3 months). The physical examination included measurement of height in cm and weight in kg, and on this basis BMI was calculated. The waist circumference was measured with tape, halfway between the lowest rib and the top of the hipbone. The hip circumference was measured with tape, at the widest part of the buttocks. The waist-to-hip ratio was calculated.

- Dual-energy X-ray absorptiometry examination

Patients underwent dual X-ray (DXA) examination using the DXA technique (Ho-logic Horizon A densitometer, Marlborough, MA, USA) to assess total body fat percentage (% fat).

- Hormonal and biochemical assays

Blood was collected in the morning while fasting.

ACTH, LH, FSH, estradiol, testosterone, DHEAS, insulin and IGF-1 concentrations were measured by the chemiluminescence immunoassay method using Immulite 2000 XPI (Siemens Healthcare Diagnostics, Erlangen, Germany). Urine free cortisol excretion was measured using a radioimmunoassay method (Immunotech, Beckman Coulter Inc., Prague, Czech Republic).

Levels of total cholesterol, triglycerides and HDL-cholesterol were measured with a colorimetric method (Alanity, Abbot, Abbott Park, IL, USA). LDL-C was calculated by means of the Friedewald equation.

Fasting plasma glucose concentrations were measured using the glucose oxidase method (Alinity, Abbott). The Homeostasis Model Assessment of Insulin Resistance (HOMA-IR) was calculated on the basis of the following formula: (Glucose mg/dL × Insulin µU/mL)/405.

- GDF15 assay

Serum samples were obtained by centrifugation of venous blood, divided into aliquots, and stored at 80 °C until use. Quantification of human GDF15 in serum was performed using a sandwich ELISA method according to the manufacturer’s protocol (BioVendor-Laboratorní medicína a.s., Brno, Czech Republic) in the presence of control samples. GDF15 concentrations were determined from a calibration curve using Gen5 version 3.10 (Bio-Tek Instruments, Inc., Winooski, VT, USA). Absorbance was measured at 450 nm, with a reference wavelength of 630 nm, using a microplate reader (EL 800 TS, Bio-Tek Instruments, Inc.). All standards, controls, and samples were analyzed in duplicate. The sensitivity of the assay was 6 pg/mL.

### 4.3. Statistical Analysis

Statistical analysis was conducted using Statistica for Windows, version 13.3 (StatSoft, Kraków, Poland). Variables were reported as mean, standard deviation (SD), median, and interquartile range (IQR). The Shapiro–Wilk test was applied to assess data normality. We used the Student’s *t*-test or Mann–Whitney test to compare quantitative variables, and the chi-square test or Fisher’s exact test for categorical variables. Correlations between parameters were determined using Pearson’s test or Spearman’s rank correlation test. A *p*-value less than 0.05 was considered statistically significant.

## Figures and Tables

**Table 1:** Characteristics of patients with primary adrenal insufficiency and controls.

	Adrenal Insufficiency (n = 37)			Control Group (n = 47)			*p*-Value
	Mean ± SD	Median	IQR	Mean ± SD	Median	IQR	
Age (years)	48.9 ±13.1	47	37–59	45.9 ± 13.7	44	34–56	0.207
Body mass (kg)	73.0 ± 15.0	71.0	61.0–80.0	69.5 ± 12.0	68.0	61.0–80.0	0.248
BMI (kg/m^2^)	25.4 ± 4.2	26.4	21.8–28.4	24.0 ± 3.4	23.4	22.0–26.0	0.113
GDF15 (pg/mL)	1276.8 ± 952.1	1021.5	768.3–1345.4	682.8 ± 270.2	626.2	486.2–838.4	**<0.001**
ACTH (pg/mL)	580.9 ± 480.5	421.0	152.0–1218.0	15.8 ± 7.7	14.0	9.6–19.6	**<0.001**
DHEAS (µmol/L)	31.6 ± 30.2	15.0	15.0–40.6	145.4 ± 70.9	148.5	99.4–196.0	**<0.001**
Fasting glucose (mg/dL)	90.8 ± 20.9	85.0	81.0–93.0	88.3 ± 6.5	88.0	84.0–92.0	0.365
HOMA-IR	44.7± 23.5	35.4	30.5–48.9	30.7 ± 10.4	28.7	24.3–35.5	0.158
Total cholesterol (mg/dL)	204.3 ± 40.8	208.0	180.0–227.0	188.0 ± 37.9	190.0	162.0–213.0	0.071
LDL cholesterol (mg/dL)	115.0 ± 35.5	116.5	91.0–129.0	108.7 ± 29.8	108.0	88.0–132.0	0.397
HDL cholesterol (mg/dL)	59.8 ± 17.9	56.0	43.0–73.0	62.9 ± 15.5	62.5	54.0–71.0	0.258
Triglycerides	133.0 ± 69.3	111.5	84.0–148.0	81.5 ± 43.9	71.0	53.0–92.0	**<0.001**
% Fat	31.8 ± 6.5	32.9	27.3–36.1	33.6 ± 4.7	33.5	30.3–37.4	0.274

IQR—interquartile range; SD—standard deviation; BMI—body mass index; GDF15—growth differentiation factor 15; ACTH—adrenocorticotropin; DHEAS—dehydroepiandrosterone sulfate; HOMA-IR—Homeostatic Model Assessment of Insulin Resistance; LDL cholesterol—low-density lipoprotein cholesterol; HDL cholesterol—high-density lipoprotein cholesterol; % Fat—percentage of fat mass in total body dual-energy X-ray absorptiometry examination; significant *p*-values are bolded.

**Table 2:** Characteristics of patients with autoimmune primary adrenal insufficiency (n = 37).

	Mean ± SD	Median	IQR
Duration of the primary adrenal insufficiency (years)	11.3 ± 12.7	6.0	1.0–20.0
Age at diagnosis	37.7 ± 12.3	36.0	29.0–42.0
Daily dose of hydrocortisone (mg)	26.3 ± 5.8	30.0	20.0–30.0
Daily dose of fludrocortisone (mg)	0.06 ± 0.06	0.05	0.025–0.1
Sodium (mmol/L)	139.2 ± 3.9	139.0	137.0–141.5
Potassium (mmol/L)	4.3 ± 0.4	4.2	4.0–4.6

SD—standard deviation; IQR—interquartile range.

**Table 3:** Correlations between GDF15 and anthropometric parameters, disease duration and laboratory results for patients with primary adrenal insufficiency (only statistically significant).

	R	*p*
Age (years)	0.534	<0.001
Duration of primary adrenal insufficiency (years)	0.364	0.027
Duration of autoimmune thyroid disease (years)	0.443	0.030
DHEAS (µmol/L)	−0.442	0.007
FSH (mIU/mL)	0.530	<0.001
LH (mIU/mL)	0.494	0.002
Estradiol (pg/mL)	−0.364	0.032
Testosterone (ng/mL)	−0.380	0.022
WHR	0.400	0.017
% Fat	0.457	0.006

DHEAS—dehydroepiandrosterone sulfate; FSH—Follicle-stimulating hormone; GDF15—growth differentiation factor 15; LH—Luteinizing hormone; WHR—waist-to-hip ratio; % Fat—percentage of fat mass in total body dual-energy X-ray absorptiometry examination; R—correlation coefficient.

**Table 4:** Correlations between GDF15 and clinical factors in controls (only statistically significant).

	R	*p*
Age (years)	0.747	<0.001
Fasting glucose (mg/dL)	0.410	0.004
IGF-1 (ng/mL)	−0.417	0.004

GDF15—growth differentiation factor 15; IGF-1—insulin-like growth factor 1; R—correlation coefficient.

## Data Availability

The original contributions presented in this study are included in the article/Supplementary Materials. Further inquiries can be directed to the corresponding author.

## Supplementary Materials

The following supporting information can be downloaded at: https://www.mdpi.com/article/10.3390/ijms27052260/s1.

## References

[1] Hsu J.Y., Crawley S., Chen M., Ayupova D.A., Lindhout D.A., Higbee J., Kutach A., Joo W., Gao Z., Fu D. (2017). Non-homeostatic body weight regulation through a brainstem-restricted receptor for GDF15. Nature.

[2] Tsai V.W.W., Husaini Y., Sainsbury A., Brown D.A., Breit S.N. (2018). The MIC-1/GDF15-GFRAL Pathway in Energy Homeostasis: Implications for Obesity, Cachexia, and Other Associated Diseases. Cell Metab..

[3] Lockhart S.M., Saudek V., O’Rahilly S. (2020). GDF15: A Hormone Conveying Somatic Distress to the Brain. Endocr. Rev..

[4] Wang D., Day E.A., Townsend L.K., Djordjevic D., Jørgensen S.B., Steinberg G.R. (2021). GDF15: Emerging biology and therapeutic applications for obesity and cardiometabolic disease. Nat. Rev. Endocrinol..

[5] Johnen H., Lin S., Kuffner T., Brown D.A., Tsai V.W.W., Bauskin A.R., Pankhurst G., Jiang L., Junankar S., Hunter M. (2007). Tumor-induced anorexia and weight loss are mediated by the TGF-β superfamily cytokine MIC-1. Nat. Med..

[6] Tsai V.W.W., Macia L., Johnen H., Kuffner T., Manadhar R., Jørgensen S.B., Lee-Ng K.K., Zhang H.P., Wu L., Marquis C.P. (2013). TGF-b Superfamily Cytokine MIC-1/GDF15 Is a Physiological Appetite and Body Weight Regulator. PLoS ONE.

[7] Tsai V.W.W., Manandhar R., Jørgensen S.B., Lee-Ng K.K.M., Zhang H.P., Marquis C.P., Jiang L., Husaini Y., Lin S., Sainsbury A. (2014). The Anorectic Actions of the TGFβ Cytokine MIC-1/GDF15 Require an Intact Brainstem Area Postrema and Nucleus of the Solitary Tract. PLoS ONE.

[8] Patel S., Alvarez-Guaita A., Melvin A., Rimmington D., Dattilo A., Miedzybrodzka E.L., Cimino I., Maurin A.C., Roberts G.P., Meek C.L. (2019). GDF15 Provides an Endocrine Signal of Nutritional Stress in Mice and Humans. Cell Metab..

[9] Coll A.P., Chen M., Taskar P., Rimmington D., Patel S., Tadross J.A., Cimino I., Yang M., Welsh P., Virtue S. (2020). GDF15 mediates the effects of metformin on body weight and energy balance. Nature.

[10] Lerner L., Tao J., Liu Q., Nicoletti R., Feng B., Krieger B., Mazsa E., Siddiquee Z., Wang R., Huang L. (2016). MAP3K11/GDF15 axis is a critical driver of cancer cachexia. J. Cachexia Sarcopenia Muscle.

[11] Kempf T., Von Haehling S., Peter T., Allhoff T., Cicoira M., Doehner W., Ponikowski P., Filippatos G.S., Rozentryt P., Drexler H. (2007). Prognostic Utility of Growth Differentiation Factor-15 in Patients With Chronic Heart Failure. J. Am. Coll. Cardiol..

[12] Breit S.N., Carrero J.J., Tsai V.W.W., Yagoutifam N., Luo W., Kuffner T., Bauskin A.R., Wu L., Jiang L., Barany P. (2012). Macrophage inhibitory cytokine-1 (MIC-1/GDF15) and mortality in end-stage renal disease. Nephrol. Dial. Transplant..

[13] Fejzo M., Rocha N., Cimino I., Lockhart S.M., Petry C.J., Kay R.G., Burling K., Barker P., George A.L., Yasara N. (2024). GDF15 linked to maternal risk of nausea and vomiting during pregnancy. Nature.

[14] Bornstein S.R., Allolio B., Arlt W., Barthel A., Don-Wauchope A., Hammer G.D., Husebye E.S., Merke D.P., Murad M.H., Stratakis C.A. (2016). Diagnosis and Treatment of Primary Adrenal Insufficiency: An Endocrine Society Clinical Practice Guideline. J. Clin. Endocrinol. Metab..

[15] Dalakas K., Allosso F., Basile C., Bergthorsdottir R., Chantzichristos D., Hessman E., Nwaru B.I., Bobbio E., Pasquali D., Johannsson G. (2025). Increased mortality in primary adrenal insufficiency: A systematic review and meta-analysis. Eur. J. Endocrinol..

[16] Melvin A., Chantzichristos D., Kyle C.J., Mackenzie S.D., Walker B.R., Johannsson G., Stimson R.H., O’Rahilly S. (2020). GDF15 Is Elevated in Conditions of Glucocorticoid Deficiency and Is Modulated by Glucocorticoid Replacement. J. Clin. Endocrinol. Metab..

[17] Engel K.B., Yamamoto K.R. (2011). The Glucocorticoid Receptor and the Coregulator Brm Selectively Modulate Each Other’s Occupancy and Activity in a Gene-Specific Manner. Mol. Cell. Biol..

[18] Chu C.C., Hsing C.H., Shieh J.P., Chien C.C., Ho C.M., Wang J.J. (2014). The cellular mechanisms of the antiemetic action of dexamethasone and related glucocorticoids against vomiting. Eur. J. Pharmacol..

[19] Tiemensma J., Andela C.D., Kaptein A.A., Romijn J.A., van der Mast R.C., Biermasz N.R., Pereira A.M. (2014). Psychological morbidity and impaired quality of life in patients with stable treatment for primary adrenal insufficiency: Cross-sectional study and review of the literature. Eur. J. Endocrinol..

[20] Hahner S., Loeffler M., Fassnacht M., Weismann D., Koschker A.C., Quinkler M., Decker O., Arlt W., Allolio B. (2007). Impaired subjective health status in 256 patients with adrenal insufficiency on standard therapy based on cross-sectional analysis. J. Clin. Endocrinol. Metab..

[21] Zdrojowy-Wełna A., Stańska A., Halupczok-Żyła J., Szcześniak D., Bolanowski M. (2023). Health-Related Quality of Life in Patients with Primary Adrenal Insufficiency. J. Clin. Med..

[22] Pfaltz M.C., Schnyder U. (2023). Allostatic Load and Allostatic Overload: Preventive and Clinical Implications. Psychother. Psychosom..

[23] Strzelec M., Szcześniak D., Zendran-Zahorska I., Kuliczkowska-Płaksej J., Słoka N., Kujawa K., Bolanowski M., Jawiarczyk-Przybyłowska A. (2025). Allostatic load index in patients with pituitary tumours: A case control study. Front. Endocrinol..

[24] Sanchis J., Ruiz V., Bonanad C., Sastre C., Ruescas A., Díaz M., Rodríguez E., Valero E., García-Blas S., Carratalá A. (2019). Growth differentiation factor 15 and geriatric conditions in acute coronary syndrome. Int. J. Cardiol..

[25] Cruz-Jentoft A.J., Sayer A.A. (2019). Sarcopenia. Lancet.

[26] Conus F., Rabasa-Lhoret R., Péronnet F. (2007). Characteristics of metabolically obese normal-weight (MONW) subjects. Appl. Physiol. Nutr. Metab. Physiol. Appl. Nutr. Metab..

[27] Roy D., Purohit P., Modi A., Khokhar M., Shukla R.K.G., Chaudhary R., Sankanagoudar S., Sharma P. (2022). Growth Differentiation Factor-15 as a Biomarker of Obese Pre-diabetes and Type 2 Diabetes Mellitus in Indian Subjects: A Case-control Study. Curr. Diabetes Rev..

[28] He X., Su J., Ma X., Lu W., Zhu W., Wang Y., Bao Y., Zhou J. (2020). The association between serum growth differentiation factor 15 levels and lower extremity atherosclerotic disease is independent of body mass index in type 2 diabetes. Cardiovasc. Diabetol..

[29] Schernthaner-Reiter M.H., Itariu B.K., Krebs M., Promintzer-Schifferl M., Stulnig T.M., Tura A., Anderwald C.H., Clodi M., Ludvik B., Pacini G. (2019). GDF15 reflects beta cell function in obese patients independently of the grade of impairment of glucose metabolism. Nutr. Metab. Cardiovasc. Dis. NMCD.

[30] Siddiqui J.A., Pothuraju R., Khan P., Sharma G., Muniyan S., Seshacharyulu P., Jain M., Nasser M.W., Batra S.K. (2022). Pathophysiological role of growth differentiation factor 15 (GDF15) in obesity, cancer, and cachexia. Cytokine Growth Factor Rev..

[31] Conte M., Ostan R., Fabbri C., Santoro A., Guidarelli G., Vitale G., Mari D., Sevini F., Capri M., Sandri M. (2019). Human Aging and Longevity Are Characterized by High Levels of Mitokines. J. Gerontol. Ser. A.

[32] Ho J.E., Mahajan A., Chen M.H., Larson M.G., McCabe E.L., Ghorbani A., Cheng S., Johnson A.D., Lindgren C.M., Kempf T. (2012). Clinical and genetic correlates of growth differentiation factor 15 in the community. Clin. Chem..

[33] Salminen A. (2024). GDF15/MIC-1: A stress-induced immunosuppressive factor which promotes the aging process. Biogerontology.

