# Supplementary material for: GDF15 in Patients with Autoimmune Primary Adrenal Insufficiency
Source: Int J Mol Sci. 2026 Feb 27;27(5):2260. doi: 10.3390/ijms27052260 (PMC12984741; doi:10.3390/ijms27052260)
Supplement: Supplementary file 1 [file ijms-27-02260-s001.zip › ijms-4136369-supplementary.pdf]

Table S1. GDF15 values in patients with primary adrenal insufficiency.

GDF15 in males with PAI, n=17 (pg/ml)	GDF15 in females with PAI, n=22 (pg/ml)
1118,4	784,1
1088,4	947,1
779,5	1278,5
741,8	586,9
1040,7	1385,6
620,4	819,428
828,4	3026,388
785,4	5418,429
1021,5	729,972
2384,2	692,616
523,7	1464,463
768,1	1345,426
670,6	1369,624
883,9	2079,197
743,4	1041,588
	3492,072
	1672,59
	768,308
	1137,168
	1236,925
	889,231
	1076,071

GDF15 - Growth Differentiation Factor 15; PAI – primary adrenal insufficiency
